# Genetic Polymorphisms in Oxidative Stress and Inflammatory Pathways as Potential Biomarkers in Alzheimer’s Disease and Dementia

**DOI:** 10.3390/antiox12020316

**Published:** 2023-01-29

**Authors:** David Vogrinc, Milica Gregorič Kramberger, Andreja Emeršič, Saša Čučnik, Katja Goričar, Vita Dolžan

**Affiliations:** 1 Pharmacogenetics Laboratory, Institute of Biochemistry and Molecular Genetics, Faculty of Medicine, University of Ljubljana, 1000 Ljubljana, Slovenia; 2 Department of Neurology, University Medical Centre Ljubljana, 1000 Ljubljana, Slovenia; 3 Faculty of Medicine, University of Ljubljana, 1000 Ljubljana, Slovenia; 4 Department of Rheumatology, University Medical Centre Ljubljana, 1000 Ljubljana, Slovenia; 5 Faculty of Pharmacy, University of Ljubljana, 1000 Ljubljana, Slovenia

**Keywords:** Alzheimer’s disease, oxidative stress, inflammation, polymorphism, biomarker

## Abstract

Oxidative stress and neuroinflammation are important processes involved in Alzheimer’s disease (AD) and mild cognitive impairment (MCI). Numerous risk factors, including genetic background, can affect the complex interplay between those mechanisms in the aging brain and can also affect typical AD hallmarks: amyloid plaques and neurofibrillary tangles. Our aim was to evaluate the association of polymorphisms in oxidative stress- and inflammation-related genes with cerebrospinal fluid (CSF) biomarker levels and cognitive test results. The study included 54 AD patients, 14 MCI patients with pathological CSF biomarker levels, 20 MCI patients with normal CSF biomarker levels and 62 controls. Carriers of two polymorphic *IL1B* rs16944 alleles had higher CSF Aβ_1–42_ levels (*p* = 0.025), while carriers of at least one polymorphic *NFE2L2* rs35652124 allele had lower CSF Aβ_1–42_ levels (*p* = 0.040). Association with *IL1B* rs16944 remained significant in the AD group (*p* = 0.029). Additionally, *MIR146A* rs2910164 was associated with Aβ_42/40_ ratio (*p* = 0.043) in AD. Significant associations with cognitive test scores were observed for *CAT* rs1001179 (*p* = 0.022), *GSTP1* rs1138272 (*p* = 0.005), *KEAP1* rs1048290 and rs9676881 (both *p* = 0.019), as well as *NFE2L2* rs35652124 (*p* = 0.030). In the AD group, *IL1B* rs1071676 (*p* = 0.004), *KEAP1* rs1048290 and rs9676881 (both *p* = 0.035) remained associated with cognitive scores. Polymorphisms in antioxidative and inflammation genes might be associated with CSF biomarkers and cognitive test scores and could serve as additional biomarkers contributing to early diagnosis of dementia.

## 1. Introduction

Age, as a complex process, is an important risk factor for neurodegeneration. Alzheimer’s disease (AD) is the most prevalent neurodegenerative disease, highly related to aging. Roughly 10–30% of people above 65 suffer from AD [1,2]. Earlier onset of clinical symptoms is uncommon and usually linked to familial genetic heritability [3]. Two major pathophysiological hallmarks of AD are deposition of amyloid β (Aβ) in neuritic plaques and accumulation of neurofibrillary tangles from tau protein. AD is often preceded by mild cognitive impairment (MCI), which is considered to be the pre-dementia stage of the disease. Severe memory and learning decline can be observed during disease progression from MCI to AD [4].

Oxidative stress is a biological process, driven by the imbalance between production of reactive oxygen species (ROS)—superoxide radical anion (O_2_^−^), hydrogen peroxide (H_2_O_2_), hydroxyl radical (HO^−^), nitric oxide (NO) and peroxynitrite (ONOO^−^)—and antioxidative defense mechanisms [5]. In the AD brain, oxidative stress can be manifested through high levels of oxidized proteins, lipoproteins and DNA damage [6,7,8]. Both AD hallmarks were also associated with ROS. Aβ fibrils can induce nitro-oxidative stress in neurons and directly increase ROS production by activating NADPH oxidase [9,10]. Furthermore, ROS can disturb normal tau function and lead to tau hyperphosphorylation [11]. The endogenous enzyme defense mechanisms, including catalase, superoxide dismutase (SOD), glutathione peroxidase and glutathione reductase, are significantly upregulated in the hippocampus and amygdala of AD brains [12]. Although not fully understood, oxidative stress can be considered as an unavoidable stage of different AD pathogeneses.

Induced oxidative stress can lead to the dysfunction of neuronal cells and contribute to neuroinflammation [13]. Driven by the immune cells in the central nervous system (CNS), the inflammatory process leads to synaptic and neuronal damage [14]. It is marked by the production of pro-inflammatory cytokines, including interleukin 1 beta (IL-1β), interleukin 6 (IL-6), interleukin 18 (IL-18) and tumor necrosis factor (TNF), chemokines, small-molecule messengers, including prostaglandins and NO, as well as ROS [15]. Deposition of Aβ and tau protein tangles is closely linked to neuroinflammation. Aβ aggregation can induce a pathogen-like recognition mechanism, guided by microglia [16,17]. The shift from beneficial microglial endocytic activity to sustained activation is a characteristic of the aged brain and neurodegeneration [18]. Upon inflammation, astrocytes in the CNS are also activated and cytokine stimulus switches their function from homeostatic to apoptotic, affecting neurons and oligodendrocytes [19,20]. Cellular crosstalk between microglia and astrocytes could, therefore, form a positive feedback loop in the AD inflammatory process, resulting in a dysregulated and self-amplifying inflammatory response [18]. After microglial recognition of Aβ, a metabolic shift occurs and a phenotypic change in microglia can be observed, leading to activation of phagocytic or inflammatory pathways [21]. The primary receptor for Aβ recognition is Toll-like receptor 2 (TLR2) that induces elevated IL-8 and TNF expression [22], while scavenger receptors upregulate IL-1β and NO production through NF-κB, c-Jun N-terminal kinase (JNK) and mitogen-activated protein kinase (MAPK) pathways [23]. In addition, the cytokines produced by microglia, including IL-1β, IL-6, TNFα and IFNγ, might enhance NF-κB-driven Aβ generation, further contributing to AD pathology [24]. Promotion of inflammatory cytokine release is also one of the key functions of macrophage migration inhibitory factor (MIF). MIF was previously associated with both Aβ and tau pathology in AD [25], but both pathogenic and protective roles have been found in different neurodegenerative diseases for MIF and its homologue DDT [26]. The effect on AD pathology was also associated with gene expression regulators such as miRNAs. Namely, the effect of *TLR2*-interacting miRNA hsa-miR-146a on Aβ has been observed in AD models [27]. Tau is also recognized as an inducer of microglial metabolic shift. Tau oligomers and fibrils facilitate microglial morphologic change and interleukin expression, especially IL-6 [28]. Microglia are capable of tau internalization [29], supposedly leading to the activation of the complement system [30]. Apart from phagocytosis, microglia facilitate tau spread through exosome secretion, contributing to AD progression [31]. Recruitment of the pyrin domain-containing 3 (NLRP3) inflammasome is also a crucial step in microglial phenotypic switch to inflammatory response, followed by caspase-1 expression and maturation of IL-1β [32]. NLRP3 activation is also a common feature between both Aβ- and tau-induced microglial response [33]. Longitudinal studies found a decrease in microglial activity in early stages of AD or MCI, followed by extensive activation with disease progression [34,35]. Although evidence suggests that there might be a protective microglial effect in MCI and opposite role in advanced AD, the exact mechanism behind the microglial dual role in neurodegeneration remains unclear.

Numerous studies have evaluated cytokine levels in body fluids of AD or mild cognitive impairment (MCI) patients. Elevated IL-1β, IL-6 and TNFα levels from peripheral blood samples were observed in AD in two large meta analyses [36,37]. Additionally, IL-6 and IL-8 were associated with lower scores on cognitive tests, such as The Mini-Mental State Exam (MMSE) [38]. The effect of inflammation on cognitive decline preceding the normal aging process is evident. However, the precise molecular processes driving it are also in the focus of AD research.

Genetic background is an important risk factor contributing to AD. Apart from genes encoding amyloid precursor protein (*APP*), presenilin-1 (*PSEN1*) and presenilin-2 (*PSEN2*) that are linked to rare familial cases of disease, polymorphisms in apolipoprotein E gene (*APOE)* are considered as the most prevalent AD risk factors in the sporadic type of disease [39,40]. In genome-wide association studies (GWAS), multiple oxidative stress and inflammation genes have also been proposed as AD risk genes [41]. Polymorphisms in *TREM2* and *IL6R* have been associated with increased AD risk [42,43,44,45], while *MEF2C* and *SPI1* were proposed as protective loci [42,46,47]. Furthermore, Aβ_42_ and tau deposition were linked to *SERPINB1*, *BCAM*, *CD33* and *IL1RAP*, all important mediators in immune response [47,48,49,50].

As several lines of evidence support the important role of oxidative stress and inflammation in dementia, including AD, our study investigated the association of common genetic polymorphisms in selected antioxidative and inflammatory genes with dementia susceptibility, AD biomarker levels and MMSE.

## 2. Materials and Methods

### 2.1. Subjects

Our study included patients with cognitive impairment as they were coming for clinical evaluation and lumbar puncture appointment at the Department of Neurology, University Medical Centre Ljubljana, Slovenia, between June 2019 and July 2021. Inclusion criteria were age above 55 and diagnosis of AD or MCI. We excluded patients with physical diseases significantly affecting cognitive performance and dementia due to diseases other than AD. Patients and their caregivers underwent a structured interview to obtain demographic and clinical data. Additional information was obtained from medical records.

The study protocol was approved by the National Medical Ethics Committee of Republic of Slovenia (0120-523/2017-4) and all the subjects provided written informed consent in accordance with the Declaration of Helsinki.

Population-based group of elderly patients without diagnosed cognitive impairment was selected as a control group. All of them participated in previous pharmacogenetic studies and provided written consent to participate in further genetic studies. Information on all diagnoses and treatments was available for all participants. Inclusion criteria were age above 65. Exclusion criteria were cancer, neurodegenerative or other neurological disease, inflammatory diseases, joint dementia or anxiety.

### 2.2. Assesment

Dementia was diagnosed using a standardized clinical assessment and patients’ history of cognitive decline. Cognitive screening was performed with the Mini-Mental State Examination (MMSE) [51]. A comprehensive diagnostic work-up, including structural brain imaging, blood laboratory tests, neuropsychological assessment and cerebrospinal fluid (CSF) dementia biomarker testing, was performed.

Patients were diagnosed as having dementia using DSM V criteria [52] after a consensus meeting with clinicians and neuropsychologists, taking into account all available information.

According to CSF biomarker levels, dementia criteria and Winblad & Peterson MCI diagnostic criteria [53], patients were stratified in three groups: AD, MCI (AD) and MCI (NOT AD). Locally validated biomarker cut-off levels were used for Aβ_42_ (>570 pg/mL), Aβ_42_/_40_ (>0.07), p-tau_181_ (<60 pg/mL) and total tau (<400 pg/mL). Patients with elevated total and p-tau_181_ and reduced Aβ_42_ and Aβ_42_/_40_ levels and with impaired daily activities were defined as AD group. Patients with MCI and AD CSF biomarker profile and normal daily functioning were included in MCI (AD) group. Patients with normal biomarker levels that had MCI and preserved daily functioning were defined as MCI (NOT AD) group.

### 2.3. Cerebrospinal Fluid Analysis

CSF was obtained via lumbar puncture between the L3/L4 and L4/L5 intervertebral space using a 25-gauge needle and collected in polypropylene tubes (Sarstedt AG & Co., Nümbrecht, Germany). CSF samples were promptly centrifuged (2000× *g*, 10 min at 20 °C), aliquoted in polypropylene tubes and stored at −80 °C until biomarker analysis was performed at the Laboratory for CSF diagnostics, Department of Neurology, University Medical Centre Ljubljana, Slovenia. Aβ_1–42_, Aβ_1–40_, p-tau_181_ and total tau were measured using the INNOTEST^®^ (Fujirebio, Europe) immunoassays, according to manufacturers’ instructions. Intra-assay variability for all biomarkers was <5%. Between-assay coefficients of variation for Aβ_1–42_, Aβ_1–40_, p-tau_181_ and total tau were 5.8%, 8.3%, 4.4% and 8.2%, respectively, as determined by the longitudinal quality control sample.

### 2.4. Genotyping

Genomic DNA was isolated using the E.Z.N.A.^®^ SQ Blood DNA Kit II (Omega Bio-tek, Inc., Norcross, GA, USA) from peripheral blood samples according to the manufacturer’s protocol. Genotyping was performed for 20 single-nucleotide polymorphisms (SNPs) in 13 genes; 5 of them are involved in oxidative stress mechanisms (*SOD2*, *CAT*, *GPX1*, *KEAP1*, *NFE2L2*), while the other 8 are important in the inflammation process (*IL1B*, *MRNA146A*, *IL6*, *TNF*, *CARD8*, *NLRP3*, *GSTP1*, *NOS1*). Polymorphism selection was performed according to published literature, as potentially functional SNPs were selected, with a minor allele frequency of at least 0.05. All of the studied SNPs were genotyped with competitive allele-specific PCR (KASP assays, LGC Biosearch Technologies, Hoddesdon, UK), according to manufacturer’s instructions.

Additionally, APOE rs7412 and rs429358 were genotyped for the assessment of *APOE4* status using real-time PCR-based Taqman assay (Applied Biosystems, Foster City, CA, USA). Combination of *APOE* rs429358 (p.Cys112Arg) and rs7412 (p.Arg158Cys) defines three polymorphic alleles, *APOE2*, *APOE3* and *APOE4*, and was used for adjustment in statistical analysis. While *APOE3* is the most common among different populations, *APOE4* is considered to significantly increase AD risk [54,55]. Ten percent of samples were genotyped in duplicate as quality control and all the results were concordant.

### 2.5. Statistical Analysis

Continuous variables were described with median and interquartile range (25–75%), while categorical variables were described with frequencies. Interquartile range was determined using weighted averages if more than two samples were included in the group and using Tukey’s hinges if two samples were included in the group. Fisher’s exact test, Mann–Whitney and Kruskal–Wallis tests were used to compare patients’ characteristics between groups. The agreement of genotype frequencies with Hardy–Weinberg equilibrium (HWE) was examined by chi-squared test. Both dominant and additive genetic models were used in the analysis. Logistic regression was used to evaluate the association of selected SNPs with binary categorical variables and to calculate the odds ratios (ORs) and their 95% confidence intervals (CIs). Fisher’s exact test was used if there were no subjects within one of the groups and for dependent categorical variables with more than two categories. Mann–Whitney or Kruskal–Wallis tests with post hoc Bonferroni corrections for pairwise comparisons were used to evaluate the association of SNPs with MMSE and biomarker levels. Bonferroni correction was used to account for multiple comparisons to decrease the chance of false-positive results. The significance threshold was set to 0.0025 and *p*-values below 0.0025 were considered statistically significant, while p-values between 0.0025 and 0.050 were considered nominally significant. IBM SPSS Statistics version 27.0 (IBM Corporation, Armonk, NY, USA) was used for all analyses. All tests were two-sided and the level of significance was set at 0.05. GraphPad Prism version 9 (GraphPad Software, LLC., San Diego, CA, USA) was used for preparation of figures.

For the comparison of genotype frequencies between cases and controls, this study had 80% power to detect ORs of approximately 2.6 or more for polymorphisms with minor allele frequency between 0.20 and 0.40, while we could detect ORs of 3.6 or more for polymorphisms with minor allele frequency of 0.10. Power calculation was conducted by the PS Power and sample size calculations, version 3.1.6 [56].

## 3. Results

### 3.1. Patients’ Characteristics

Our study included 150 subjects: 88 patients with memory deficits, of which 54 were AD patients, 14 MCI patients with pathological CSF biomarker levels (MCI (AD)), 20 MCI patients with normal CSF biomarker levels (MCI (NOT AD)) and 62 population-based controls without diagnosed cognitive impairment. Median age of all dementia patients at enrolment was 77 (72.25–80) years and differed significantly from controls (69.2 (66.48–73.18), *p* < 0.001). There were more female subjects in both groups (dementia patients: 56.8%, controls: 66.1%, *p* = 0.309). Clinical characteristics of all patients with dementia and of individual groups (AD, MCI (AD) and MCI (NOT AD)) are summarized in Table 1. AD patients were significantly older compared to patients with MCI (AD) and MCI (NOT AD) (*p* = 0.010). *APOE4* carriers were most frequent in the AD group, but differences were not significant (*p* = 0.138). Significant differences in all CSF biomarker levels (Aβ, Aβ_42/40_, total tau and p-tau_181_) were observed between groups (all *p* < 0.001). AD patients also achieved significantly lower results in the cognitive test (*p* < 0.001).

Genotype frequencies of all 20 investigated SNPs in *SOD2*, *CAT*, *GPX1*, *IL1B*, *MIR146A*, *IL6*, *TNF*, *CARD8*, *NLRP3*, *GSTP1*, *NOS1*, *KEAP1* and *NFE2L2* genes in the whole cohort are presented in Table S1. Genotype frequencies of *NOS1* rs2293054, *KEAP1* rs9676881 and *NFE2L2* rs6706649 were not in agreement with HWE in the control group. However, due to the fact that the control population was not randomly selected, we included those genes in further analysis.

### 3.2. Association of Investigated SNPs with Dementia and AD Susceptibility

A comparison of genotype frequencies between all dementia patients and controls is presented in Table 2. Subjects who were heterozygous for *TNF* rs1800629 polymorphism were less likely to have dementia (*p* = 0.017, OR = 0.368 (0.163–0.834)). The protective association with dementia susceptibility remained nominally significant, even after adjustment for age and *APOE* carrier status (*p* = 0.011, OR = 0.274 (0.101–0.741)). In the dominant model, the association was also nominally significant, both in univariable and multivariable analysis (both *p* = 0.049, Table 2). On the other hand, all carriers of two *NOS1* rs2293054 G alleles had dementia (*p* = 0.019).

Genotype distribution of investigated polymorphisms in different dementia pathologies is presented in Table S2. At least one polymorphic *NFE2L2* rs35652124 C allele was more common in AD compared to both MCI groups (*p* = 0.021). No significant or nominally significant differences were observed for other investigated SNPs.

Additionally, we compared genotype frequencies of only AD patients with controls (Table S3). Carriers of one polymorphic *IL1B* rs16944 C allele tended to have lower risk for AD (*p* = 0.049, OR = 0.315 (0.099–0.997)). Association remained nominally significant, even after the adjustment for age and *APOE* carrier status (*p* = 0.039, OR = 0.206 (0.046–0.924)). Carriers of two polymorphic *NOS1* rs2293054 G alleles were present among AD patients only (*p* = 0.012). Carriers of at least one polymorphic *TNF* rs1800629 allele were less likely to have AD, when adjusting for age and *APOE* carrier status (*p* = 0.025, OR = 0.246 (0.072–0.840)).

### 3.3. Association of Investigated SNPs with CSF Biomarker Levels and MMSE

**Figure 1:**
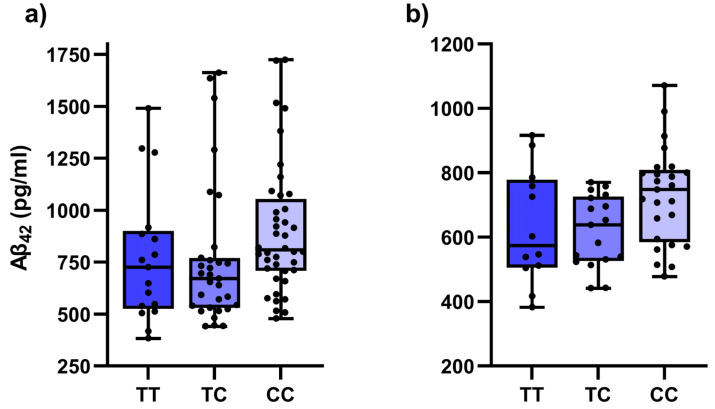
Comparison of Aβ_42_ cerebrospinal fluid levels between different *IL1B* rs16944 genotypes in: (**a**) whole patient cohort; (**b**) Alzheimer’s disease patients only.

Among investigated SNPs, three were associated with different CSF biomarkers among all patients with dementia (Table 3). Carriers of two polymorphic *IL1B* rs16944 C alleles had higher Aβ levels (*p* = 0.020) (Figure 1a). On the other hand, decreased Aβ levels were observed in carriers of one polymorphic *NFE2L2* rs35652124 C allele (*p* = 0.031). The association did not reach statistical significance or nominal significance in the dominant model (*p* = 0.053). For *NFE2L2* rs6721961, decreased total tau was observed in carriers of at least one polymorphic T allele (*p* = 0.020).

The association of *IL1B* rs16944 C allele with Aβ remained nominally significant when only patients with AD were included in the analysis (Figure 1b, Table S4); carriers of two polymorphic C alleles had higher Aβ levels (*p* = 0.038). Additionally, carriers of at least one polymorphic C *MIR146A* rs2910164 allele had higher Aβ_42/40_ ratio (*p* = 0.043).

**Figure 2:**
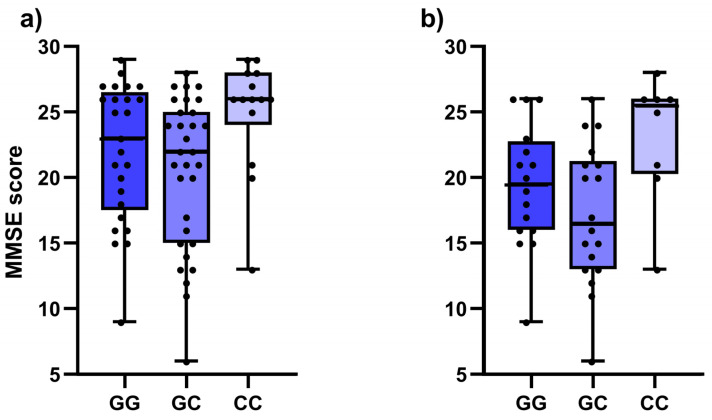
Comparison of the Mini-Mental State Exam (MMSE) scores between different *KEAP1* rs1048290 genotypes in: (**a**) whole patient cohort; (**b**) Alzheimer’s disease patients only.

Nominally significant associations with the MMSE cognitive test were observed in the dementia group for five of the selected SNPs (Table 4). Higher test scores were observed in carriers of two polymorphic alleles for *CAT* rs1001179 (*p* = 0.022), *KEAP1* rs1048290 (*p* = 0.019, Figure 2a) and *KEAP1* rs9676881 (*p* = 0.019). Furthermore, carriers of at least one polymorphic *GSTP1* rs1138272 T allele had higher test scores (*p* = 0.005). Conversely, polymorphic *NFE2L2* rs35652124 C allele was associated with lower test scores in both the additive (*p* = 0.030) and dominant model (*p* = 0.024). In AD patients, lower cognitive test scores were observed in patients with the heterozygous *IL1B* rs1071676 genotype (*p* = 0.004), while carriers of two polymorphic *KEAP1* rs1048290 alleles (*p* = 0.035, Figure 2b) and *KEAP1* rs9676881 (*p* = 0.035) had higher MMSE scores (Table 4).

## 4. Discussion

Our study evaluated the effect of selected SNPs from oxidative stress and inflammation pathways on the risk for AD and assessed their associations with CSF biomarkers and cognitive test results. Among inflammatory genes, polymorphisms in *IL1B* and *TNF* showed a protective function in the development of dementia or AD, while *MIR146A* was associated with CSF biomarkers. Observed associations of polymorphisms in *CAT*, *GSTP1*, *NFE2L2* and *KEAP1* with dementia support the importance of antioxidative mechanisms in AD.

Several studies evaluating the effect of genetic polymorphisms in the oxidative stress- and inflammation-related pathways on AD risk are summarized in multiple meta-analyses [57,58,59]. Although the association of genetic variability with most prominent AD CSF biomarkers (Aβ_42_, total-tau, P-tau_181_) is readily studied, oxidative stress- and inflammation-related genes are usually overlooked. Only a couple of studies assessed the association of polymorphisms in cytokine-encoding genes with AD biomarker levels [60,61,62]. To see a broader picture of the influence of oxidative stress and inflammation pathways on AD genetic background, we selected a comprehensive list of SNPs and assessed their correlations with disease pathology.

Among antioxidative genes, *CAT*, *GSTP1*, *NOS1*, *NFE2L2*, and *KEAP1* were associated with dementia or AD. *CAT* and *GSTP1* genetic variability was thoroughly studied in oxidative-stress-related diseases, especially different types of cancer, while in neurodegeneration, *NFE2L2* and *KEAP1* have recently gained the interest of researchers. On the other hand, no significant association in *NOS1* on AD susceptibility is published to date.

We observed an association of *CAT* rs1001179 with MMSE in all patients with dementia. To date, no *CAT* polymorphisms are associated with AD or MCI [63]. Catalase is a ubiquitous antioxidative enzyme, catalyzing the reductions of hydrogen peroxide to water. Aβ can cause an accumulation of hydrogen peroxide, since Aβ-driven reductions in catalase activity have been observed [64,65], thus, promoting oxidative stress in AD [66]. The *CAT* rs1001179 polymorphism leads to alterations in the transcription factor binding site at the promoter region and affects catalase blood levels [67]. Although previous work found no significant effect of *CAT* rs1001179 on AD risk [63], our association between MMSE and rs1001179 implies catalase might contribute to dementia.

Observed association of *GSTP1* rs1138272 with higher MMSE scores is, to the best of our knowledge, a novel finding. *GSTP1* rs1138272 is a missense polymorphism that can alter catalytic activity of glutathione S-transferase (GST) in oxidative stress [68,69]. GST gene family is important in glutathione-induced detoxification mechanisms, preventing oxidative damage of biomolecules [70,71]. Increased levels of GST pi (π), encoded by *GSTP1*, were found in an AD mouse model [72]. Moreover, increased activity of both catalase and GSTs has been found in CSF [73,74,75] and blood samples [76] of patients with different dementia types. Previously, the *GSTP1* rs1695 polymorphism was associated with increased AD risk in different studies and one meta-analysis [58,77,78]. Association of *GSTP1* rs1695 with MMSE was also reported [79], but these results were not confirmed in our study.

The presence of the *NOS1* rs2293054 G allele only in patients with dementia or AD, in our study, was a surprising outcome. *NOS1* encodes the neuronal isoform of the nitric oxide synthase that is the main source of brain nitric oxide (NO) [80]. Although *NOS1* rs2293054 has not yet been studied in AD, association with clinical phenotypes of ischemic stroke [81] and Parkinson’s disease treatment [82] was observed. In accordance with our results, higher AD risk was found in another functional *NOS1* promoter polymorphism in two independent cohorts [83,84]. Although the investigated antioxidative enzymes were not associated with CSF biomarker levels in our study, they could serve as additional biomarkers of dementia.

Multiple effects of *NFE2L2* and *KEAP1* on dementia and AD were found in our study. An association with Aβ_42_ levels and MMSE scores was found for *NFE2L2* rs35652124 in the combined group with dementia. *NFE2L2* rs6721961 was also associated with tau levels in AD and MCI. Additionally, we found association with MMSE in *KEAP1* rs1048290 and rs9676881, both in the whole cohort as well as in AD patients only. Nuclear factor E2-related factor 2 (NRF2, encoded by *NFE2L2* gene) and kelch-like ECH-associating protein 1 (KEAP1) are two of the main regulators of redox balance, crucial for human stress response. They are involved in response to oxidative stress, but also important in metabolism of xenobiotics and inflammatory response (reviewed in [13]). Consistent with our results, different studies investigating *NFE2L2* and *KEAP1* polymorphisms found no association with AD risk; however, one *NFE2L2* haplotype was linked to faster disease progression [85]. In the same study, no association with CSF biomarkers and MMSE in AD patients was observed, which is in contrast with our findings. NRF2 is aberrantly expressed in different brain cells, with an observed age-associated decrease [86] and reduced expression in AD patients [87]. NRF2 deficit was associated with AD pathology: NRF2 knockout in mice leads to an increase in Aβ and tau levels [88,89] and worsens cognitive decline [90]. The NRF2-KEAP1 signaling axis is thoroughly studied in neurodegeneration, with the focus on potential therapeutic application [13,91,92]. Our observed associations for NRF2 and KEAP1 support the potential role of the NRF2-KEAP1 axis in neurodegeneration and could partially explain the missing connection between oxidative stress and neurodegeneration.

In the present study, the effect of *IL1B*, *MIR146A* and *TNF* on dementia or AD susceptibility confirmed previously published data [27,60,61,62]. We observed a protective effect of the *TNF* rs1800629 A allele in a combined group of all patients with AD or MCI, while the results did not reach significance in the AD group only. TNF-α is one of the most studied inflammatory cytokines in AD as TNF-α plays a central role in the cytokine cascade during microglial activation. Subsequently, TNF-α can induce neuronal death by activating TNF receptor 1 if the NF-κB pathway is inhibited [93]. *TNF* rs1800629 polymorphism is part of the promoter region and altered expression of TNF-α and soluble TNF receptors has been linked to *TNF* rs1800629 and other promoter polymorphisms [94,95,96,97,98]. Previously, the protective role of this polymorphism was observed in a Finnish population [61] but was not confirmed in another independent cohort [60]. Consistent with our results, no differences among AD and MCI were observed [99]. TNF-α levels in blood and CSF were associated with MCI and AD [100], and elevated TNF-α appears to correlate with disease progression [101]. However, the results regarding the association of *TNF* rs1800629 with biomarker levels differ among studies: *TNF* rs1800629 was previously associated with AD CSF Aβ_42_ and P-tau_231_ in some studies [61,62], while no association was observed in other studies [60,102], similarly to our results, suggesting further studies are needed to evaluate the association of this polymorphism with AD biomarkers.

In our study, *IL1B* polymorphisms were associated with AD risk, pathology and cognitive decline. *IL1B* rs16944 genotype frequencies tended to differ between AD patients and controls. The association between *IL1B* rs16944 and higher Aβ_42_ levels was observed in both the all dementia and AD group. Additionally, the heterozygous *IL1B* rs1071676 genotype was associated with lower MMSE scores in AD. IL-1β is another cytokine that has been extensively studied in AD. IL-1β induces synaptic loss by simultaneously activating multiple pathways that require both pre- and post-synaptic activity [103]. Different *IL1B* polymorphisms can affect IL-1β expression [104,105,106]. *IL1B* rs16944 is located in the promoter region and can affect IL-1β expression, while rs1071676 in 3′ untranslated region of *IL1B* gene could modify miRNA binding. Selected SNPs did not reach significant associations with AD patients in any previous studies, including meta-analyses [59,107,108,109]. However, increased CSF p-tau and t-tau levels were found in AD carriers of the *IL1B* rs1143623 polymorphic G allele [62], which was not replicated in our study. With that in mind, our results add to the importance of *IL1B* genetic variability in AD or dementia.

MiR-146a is another important factor for inflammation, interacting with IL-1β and TNF-α [110]. An inhibitory function of miR-146a on IL-1β secretion through IRAK1 was found [111,112]. In our study, an SNP in the coding region of primary hsa-miR-146a, *MIR146A* rs2910164, was associated with higher Aβ_42/40_ ratio in carriers of at least one polymorphic allele. The rare C allele was previously linked to increased AD risk and it was proposed that it could affect binding to miRNA target genes [27]. Consistent with our results, *MIR146A* rs2910164 was not associated with MMSE scores [113]. On the other hand, a different *MIR146A* polymorphism, rs57095329, was more important for miR-146a expression and AD susceptibility in the same study. To the best of our knowledge, none of the studies investigated the association of this polymorphism with CSF biomarkers. However, the expression of miR-146a was altered in AD patients [114,115,116]. Taken together, these findings support the important interplay between cytokines (TNF-α, IL-1β) and regulatory elements (miRNA-146a) in neuroinflammation.

Our study has some limitations. The sample size was relatively small and some clinical parameters, especially cognitive test scores, were not available for all patients. The smaller sample size is partly due to the fact that the patients were included during their lumbar punction appointment to assess CSF biomarkers. Although it is difficult to detect the contributions of many factors in a study with a smaller sample size, a similar effect can occur due to phenotypic heterogeneity in larger studies as well. We also accounted for multiple comparisons in the statistical analysis. Another limitation was the observed deviation from HWE for some SNPs in the control group that can be partially explained with the non-random selection of patients. A larger and more homogenous control group would help in overcoming that issue. On the other hand, our study had several strengths. All the patients were recruited from the same department and evaluated according to the same protocol. We comprehensively assessed the simultaneous influence of several clinical and genetic parameters on AD risk and pathology. We were the first to assess the genetic variability in oxidative stress and inflammatory pathways among Slovenian patients. Furthermore, only a few studies focused on the association of selected genes with CSF biomarkers. Usually, a single gene effect on AD pathology is of interest, so our pathway-based approach helps to elucidate the broader picture of disease mechanisms.

We were among the first to highlight the potential of the KEAP1-NRF2 axis in AD and dementia, opening up a new window with therapeutic potential. Due to the versatile role of the KEAP1-NRF2 axis in numerous diseases with oxidative stress and inflammation as underlying pathological features, the protective effects of NRF2 have been a research focus lately [117]. In the field of autoimmune diseases, NRF2-activator dymethyl fumarate is already approved for use in psoriasis and MS. However, activation of NRF2 could be one of the promising candidates in a multi-target therapy approach in AD. To date, several NRF2 activators for AD treatment have advanced to preclinical studies in mouse AD models and clinical trials [118]. Genetic variability in this pathway could also contribute to a better understanding of the mechanisms underlying the KEAP1-NRF2 axis in the search for suitable therapy approaches.

## 5. Conclusions

Our data suggest that genetic variability in oxidative stress- and inflammation-related genes might affect susceptibility for AD and MCI, and may be associated with CSF biomarkers levels and cognitive test scores. Observed associations support the important role of oxidative stress and neuroinflammation pathways in AD pathogenesis and could contribute to a better understanding of dementia and help identify additional biomarkers contributing to early diagnosis of cognitive decline.

## Figures and Tables

**Table 1:** Clinical characteristics of all patients with dementia (*N* = 88) and of patients with AD (*N* = 54), MCI (AD) (*N* = 14) and MCI (NOT AD) diagnosis (*N* = 20).

Characteristic	Category/Unit	Dementia	AD	MCI (AD)	MCI (NOT AD)	*p*
Sex	Male, N (%)	38 (43.2)	23 (42.6)	4 (28.6)	11 (55)	0.326 ^a^
	Female, N (%)	50 (56.8)	31 (57.4)	10 (71.4)	9 (45)	
Age	Years, median (25–75%)	77 (72.25–80)	78 (74.75–81)	76.5 (72.25–79.25)	71.5 (67–77.5)	0.010 ^b^
Education	Years, median (25–75%)	12 (8–12.25) [2]	11 (8–12) [1]	12 (11.5–15.5) [1]	11.5 (8–14.5)	0.156 ^b^
Height	cm, median (25–75%)	168 (160.50–172) [11]	168 (160–172) [8]	162.5 (160.3–168.8) [2]	172 (163–175) [1]	0.333 ^b^
Weight	kg, median (25–75%)	68 (57–79) [11]	63 (55–77) [7]	70 (54–79) [3]	70 (62–85) [1]	0.153 ^b^
BMI	kg/m^2^, median (25–75%)	25 (21–28) [13]	24.5 (21–27) [9]	27 (20.6–29.2) [3]	26.3 (22.6–28.6) [1]	0.151 ^b^
APOE status	*APOE4* carriers, N (%)	39 (44.3)	29 (53.7)	5 (35.7)	5 (25)	0.138 ^a^
MMSE	Score, median (25–75%)	24 (17.8–26) [18]	20 (15–24) [12]	26 (25–27) [3]	27 (24.5–27.5) [3]	<0.001 ^b^
Aβ_42_	pg/mL, median (25–75%)	745 (570–964.5) [2]	688 (538.5–772) [1]	740.5 (556–894.8)	1297 (1091–1540) [1]	<0.001 ^b^
Aβ_42/40_ ratio	Median (25–75%)	0.06 (0.05–0.08) [8]	0.06 (0.04–0.06) [3]	0.06 (0.03–0.07) [2]	0.11 (0.09–0.13) [3]	<0.001 ^b^
Total tau	pg/mL, median (25–75%)	605 (466.3–884.3) [2]	778 (573.5–991) [1]	557 (467.8–902.3)	320 (243–404) [1]	<0.001 ^b^
p-tau	pg/mL, median (25–75%)	88.5 (72.8–122.3) [2]	98 (82.5–128) [1]	86.5 (73–127.3)	53 (43–72) [1]	<0.001 ^b^

AD: Alzheimer’s disease; BMI: body mass index; MCI: mild cognitive impairment; MMSE: The Mini-Mental State Exam. ^a^ Fisher’s exact test; ^b^ Kruskal-Wallis test.

**Table 2:** Comparison of genotype frequencies among patients with dementia and controls.

Gene	SNP	Genotype	Controls N (%)	Dementia N (%)	OR (95% CI)	*p*	OR_adj_ (95% CI)	P_adj_
*SOD2*	rs4880	CC	15 (24.2)	20 (22.7)	Reference		Reference	
		CT	29 (46.8)	46 (52.3)	1.19 (0.527–2.688)	0.676	1.538 (0.604–3.917)	0.366
		TT	18 (29)	22 (25)	0.917 (0.367–2.287)	0.852	1.037 (0.366–2.937)	0.945
		CT + TT	47 (75.8)	68 (77.3)	1.085 (0.505–2.334)	0.834	1.336 (0.558–3.201)	0.558
*CAT*	rs1001179	CC	38 (61.3)	61 (69.3)	Reference		Reference	
		CT	19 (30.6)	21 (23.9)	0.689 (0.328–1.445)	0.324	1.245 (0.312–4.966)	0.756
		TT	5 (8.1)	6 (6.8)	0.748 (0.213–2.62)	0.649	0.789 (0.181–3.433)	0.753
		CT + TT	24 (38.7)	27 (30.7)	0.701 (0.354–1.387)	0.308	1.498 (0.691–3.244)	0.306
*GPX1*	rs1050450	CC	29 (46.8)	42 (47.7)	Reference		Reference	
		CT	27 (43.5)	37 (42)	0.946 (0.477–1.878)	0.874	0.966 (0.445–2.099)	0.931
		TT	6 (9.7)	9 (10.2)	1.036 (0.332–3.226)	0.952	1.212 (0.330–4.457)	0.772
		CT + TT	33 (53.2)	46 (52.2)	0.962 (0.502–1.846)	0.962	1.009 (0.485–2.101)	0.980
*IL1B*	rs1143623	GG	36 (58.1)	49 (55.7)	Reference		Reference	
		GC	21 (33.9)	25 (28.4)	0.875 (0.425–1.801)	0.716	0.789 (0.353–1.767)	0.565
		CC	5 (8.1)	14 (15.9)	2.057 (0.679–6.23)	0.202	1.610 (0.443–5.852)	0.470
		GC + CC	26 (42)	39 (43.3)	1.102 (0.571–2.125)	0.772	0.935 (0.444–1.968)	0.860
	rs16944	TT	6 (9.7)	17 (19.3)	Reference		Reference	
		TC	27 (43.5)	31 (35.2)	0.405 (0.14–1.174)	0.096	0.462 (0.140–1.524)	0.205
		CC	29 (46.8)	40 (45.5)	0.487 (0.171–1.386)	0.177	0.631 (0.194–2.057)	0.445
		TC + CC	56 (90.3)	71 (80.7)	0.447 (0.166–1.21)	0.113	0.545 (0.178–1.665)	0.287
	rs1071676	CC	37 (59.7)	50 (56.8)	Reference		Reference	
		CG	18 (29)	31 (35.2)	1.274 (0.621–2.618)	0.509	1.176 (0.533–2.597)	0.687
		GG	7 (11.3)	7 (8)	0.74 (0.239–2.292)	0.602	0.638 (0.171–2.379)	0.503
		CG + GG	25 (40.3)	38 (43.2)	1.125 (0.581–2.176)	0.727	1.037 (0.497–2.164)	0.922
*MIR146A*	rs2910164	GG	38 (61.3)	51 (58)	Reference		Reference	
		GC	21 (33.9)	30 (34.1)	1.064 (0.53–2.139)	0.861	1.058 (0.485–2.307)	0.887
		CC	3 (4.8)	7 (8)	1.739 (0.422–7.166)	0.444	2.544 (0.466–13.88)	0.281
		GC + CC	24 (38.7)	37 (42)	1.149 (0.592–2.23)	0.682	1.191 (0.565–2.512)	0.646
*IL6*	rs1800795	GG	21 (33.9)	32 (36.4)	Reference		Reference	
		GC	27 (43.5)	43 (48.9)	1.045 (0.503–2.171)	0.906	1.16 (0.503–2.672)	0.728
		CC	14 (22.6)	13 (14.8)	0.609 (0.239–1.551)	0.299	0.627 (0.215–1.830)	0.393
		GC + CC	41 (66.1)	56 (63.6)	0.896 (0.453–1.773)	0.753	0.973 (0.447–2.117)	0.945
*TNF*	rs1800629	GG	42 (67.7)	72 (81.8)	Reference		Reference	
		GA	19 (30.6)	12 (13.6)	0.368 (0.163–0.834)	**0.017**	0.274 (0.101–0.741)	**0.011**
		AA	1 (1.6)	4 (4.5)	2.333 (0.252–21.57)	0.455	5.445 (0.451–65.75)	0.182
		GA + AA	20 (32.2)	16 (18.1)	0.467 (0.218–0.997)	**0.049**	0.398 (0.159–0.996)	**0.049**
*CARD8*	rs2043211	AA	32 (51.6)	37 (42)	Reference		Reference	
		AT	26 (41.9)	40 (45.5)	1.331 (0.671–2.637)	0.413	1.074 (0.490–2.354)	0.858
		TT	4 (6.5)	11 (12.5)	2.378 (0.689–8.205)	0.170	1.977 (0.515–7.585)	0.321
		AT + TT	30 (48.4)	51 (57.7)	1.47 (0.765–2.827)	0.248	1.202 (0.569–2.536)	0.630
*NLRP3*	rs35829419	CC	58 (93.5)	85 (96.6)	Reference		Reference	
		CA	4 (6.5)	3 (3.4)	0.512 (0.11–2.372)	0.392	0.560 (0.098–3.208)	0.515
*GSTP1*	rs1695	AA	28 (45.2)	36 (40.9)	Reference		Reference	
		AG	28 (45.2)	38 (43.2)	1.056 (0.527–2.114)	0.879	1.279 (0.583–2.806)	0.540
		GG	6 (9.7)	14 (15.9)	1.815 (0.619–5.325)	0.278	1.731 (0.523–5.729)	0.369
		AG + GG	34 (54.9)	52 (59.1)	1.19 (0.617–2.293)	0.604	1.366 (0.648–2.877)	0.412
	rs1138272	CC	51 (82.3)	72 (81.8)	Reference		Reference	
		CT + TT	11 (17.7)	16 (18.2)	1.03 (0.442–2.404)	0.945	0.869 (0.338–2.236)	0.772
*NOS1*	rs2293054	AA	37 (59.7)	44 (50)	Reference		Reference	
		AG	25 (40.3)	36 (40.9)	1.211 (0.618–2.371)	0.577	1.104 (0.515–2.364)	0.800
		GG	0	8 (9.1)	/	**0.019** *	/	/
		AG + GG	25 (40.3)	44 (50)	1.48 (0.767–2.856)	0.242	1.409 (0.674–2.944)	0.362
	rs2682826	GG	32 (51.6)	40 (45.5)	Reference		Reference	
		GA	26 (41.9)	39 (44.3)	1.2 (0.608–2.369)	0.599	0.985 (0.456–2.128)	0.969
		AA	4 (6.5)	9 (10.2)	1.8 (0.507–6.385)	0.363	0.932 (0.230–3.782)	0.922
		GA + AA	30 (48.4)	48 (54.5)	1.28 (0.667–2.455)	0.458	0.976 (0.466–2.044)	0.950
*KEAP1*	rs1048290	GG	21 (33.9)	29 (33)	Reference		Reference	
		GC	36 (58.1)	41 (46.6)	0.825 (0.402–1.691)	0.599	0.582 (0.253–1.340)	0.203
		CC	5 (8)	18 (20.5)	2.607 (0.835–8.142)	0.099	1.947 (0.534–7.105)	0.313
		GC + CC	41 (66.1)	59 (67)	1.042 (0.523–2.075)	0.907	0.732 (0.328–1.631)	0.445
	rs9676881	GG	20 (32.2)	30 (34.1)	Reference		Reference	
		GA	37 (59.7)	40 (45.5)	0.721 (0.35–1.482)	0.373	0.540 (0.234–1.243)	0.147
		AA	5 (8)	18 (20.5)	2.4 (0.767–7.512)	0.133	1.851 (0.506–6.765)	0.352
		GA + AA	42 (67.7)	58 (66)	0.921 (0.461–1.838)	0.815	0.68 (0.305–1.519)	0.347
*NFE2L2*	rs6706649	CC	50 (80.6)	68 (77.3)	Reference		Reference	
		CT	9 (14.5)	18 (20.5)	1.471 (0.61–3.544)	0.390	1.266 (0.476–3.369)	0.637
		TT	3 (4.8)	2 (2.3)	0.49 (0.079–3.044)	0.444	0.336 (0.044–2.557)	0.292
		CT + TT	12 (19.3)	20 (22.8)	1.225 (0.549–2.737)	0.620	1.021 (0.415–2.511)	0.963
	rs6721961	GG	48 (77.4)	65 (73.9)	Reference		Reference	
		GT + TT	14 (22.6)	23 (26.1)	1.213 (0.566–2.599)	0.619	1.312 (0.557–3.09)	0.535
	rs35652124	TT	23 (37.1)	37 (42)	Reference		Reference	
		TC	26 (41.9)	39 (44.3)	0.932 (0.454–1.914)	0.849	0.689 (0.3–1.584)	0.381
		CC	13 (21)	12 (13.6)	0.574 (0.224–1.471)	0.248	0.530 (0.187–1.508)	0.234
		TC + CC	39 (62.9)	51 (57.9)	0.813 (0.417–1.584)	0.543	0.633 (0.296–1.357)	0.240

* calculated using Fisher’s exact test. Adj: adjusted for APOE status and age; CI: confidence interval; OR: odds ratio; SNP: single-nucleotide polymorphism. Statistically significant results are printed in bold.

**Table 3:** Association of investigated polymorphisms with cerebrospinal fluid biomarkers among all patients with dementia.

SNP	Genotype	Aβ_42_ (pg/mL)	*p*	Aβ_42_/_40_ Ratio	*p*	Total tau (pg/mL)	*p*	pTau (pg/mL)	*p*
*SOD2* rs4880	CC	764.5 (640.3–1032.5)	0.104	0.06 (0.05–0.08)	0.059	558 (491.5–770.5)	0.523	85.5 (69.8–102.5)	0.263
	CT	786.5 (600–1023)		0.06 (0.05–0.09)		601.5 (403.8–895)		86.5 (61–123)	
	TT	624.5 (536–799.8)		0.05 (0.04–0.07)		615 (486.5–1064)		99 (76.3–148)	
	CT + TT	740.5 (563.3–952.3)	P_dom_ = 0.758	0.06 (0.04–0.08)	P_dom_ = 0.501	611 (461–897)	P_dom_ = 0.683	88.5 (70.5–123.8)	P_dom_ = 0.530
*CAT* rs1001179	CC	747 (553.5–917.5)	0.372	0.06 (0.05–0.08)	0.737	601 (462–879.5)	0.891	87 (71–123)	0.835
	CT	731 (580.5–1030.5)		0.06 (0.05–0.07)		594 (465.5–883)		88 (72–111.5)	
	TT	913.5 (742.5–1076.5)		0.06 (0.03–0.11)		586.5 (279.8–903.8)		84 (42.3–140.3)	
	CT + TT	759 (591–1005)	P_dom_ = 0.486	0.06 (0.05–0.08)	P_dom_ = 0.435	594 (461–894)	P_dom_ = 0.825	88 (70–114)	P_dom_ = 0.562
*GPX1* rs1050450	CC	759 (597–957)	0.793	0.06 (0.05–0.08)	0.401	601.5 (482.8–836)	0.974	92.5 (75.8–123.5)	0.771
	CT	743 (542–1082)		0.06 (0.05–0.08)		601 (429.5–904.5)		87 (59–119.5)	
	TT	747 (592.5–852)		0.06 (0.05–0.06)		567 (473.5–858)		84 (72–116.5)	
	CT + TT	745 (564–964.5)	P_dom_ = 0.581	0.06 (0.05–0.08)	P_dom_ = 0.932	584 (459.8–895)	P_dom_ = 0.854	85.5 (66.3–118.3)	P_dom_ = 0.525
*IL1B* rs1143623	GG	795 (626–997.5)	0.154	0.06 (0.05–0.08)	0.684	617 (471.5–887.5)	0.760	89 (75.5–116.5)	0.863
	GC	688 (534.5–1182)		0.06 (0.05–0.09)		549 (401.5–871)		84 (60.5–126.5)	
	CC	686 (509.3–866.3)		0.06 (0.05–0.08)		596.5 (493.5–900.3)		95.5 (68–118.5)	
	GC + CC	688 (530–885)	P_dom_ = 0.070	0.06 (0.05–0.08)	P_dom_ = 0.475	567 (404–878)	P_dom_ = 0.650	87 (68–123)	P_dom_ = 0.644
*IL1B* rs16944	TT	725 (524.5–900.5)	0.020	0.06 (0.05–0.08)	0.071	547 (435.5–875)	0.573	79 (64.5–115.5)	0.293
	TC	670 (530–770)		0.06 (0.04–0.07)		613 (470–1013)		98 (72–128)	
	CC	808.5 (708–1054.5)		0.06 (0.06–0.09)		582.5 (457.3–874.5)		81 (74.3–110.8)	
	TC + CC	748 (582–990)	P_dom_ = 0.369	0.06 (0.05–0.08)	P_dom_ = 0.920	609 (461–894)	P_dom_ = 0.627	88 (74–123)	P_dom_ = 0.428
*IL1B* rs1071676	GG	723 (557.3–917.3)	0.337	0.06 (0.04–0.08)	0.414	611 (473.8–885.3)	0.672	93.5 (73.8–125.8)	0.398
	GC	788 (570–1077)		0.06 (0.05–0.08)		544 (404–894)		81 (61–114)	
	CC	799 (711–990)		0.07 (0.06–0.09)		709 (400–855)		95 (60–111)	
	GC + CC	793.5 (627–1072.5)	P_dom_ = 0.179	0.06 (0.06–0.08)	P_dom_ = 0.430	546.5 (403–864.8)	P_dom_ = 0.386	82.5 (60.8–111.8)	P_dom_ = 0.200
*MIR146A* rs2910164	GG	748 (561–1005)	0.748	0.06 (0.05–0.08)	0.994	525 (454–881)	0.556	81 (70–117)	0.728
	GC	714.5 (570–893)		0.06 (0.04–0.07)		617 (509–901.3)		92 (73–123.5)	
	CC	795 (638–1297)		0.05 (0.05–0.09)		722 (324–872)		93 (50–123)	
	GC + CC	718 (576–915)	P_dom_ = 0.735	0.06 (0.05–0.08)	P_dom_ = 0.943	617 (497–886)	P_dom_ = 0.310	93 (72–122.5)	P_dom_ = 0.465
*IL6* rs1800795	GG	745 (579.8–816.3)	0.407	0.06 (0.05–0.07)	0.402	582.5 (471–902.8)	0.832	87.5 (77–120.8)	0.849
	GC	759 (570–1087)		0.06 (0.05–0.09)		601 (404–894)		87 (61–114)	
	CC	669 (538.5–1081)		0.06 (0.04–0.11)		609 (386–837)		92 (63.5–126)	
	GC + CC	758.5 (570–1083.3)	P_dom_ = 0.225	0.06 (0.05–0.09)	P_dom_ = 0.178	605 (418.3–878.8)	P_dom_ = 0.690	88 (62.8–120.8)	P_dom_ = 0.579
*TNF* rs1800629	GG	753 (570–952.3)	0.881	0.06 (0.05–0.08)	0.309	615 (462.8–907.8)	0.246	92 (70.5–123.8)	0.354
	GA	745 (594.5–863.8)		0.07 (0.05–0.10)		555.5 (471–704)		88 (71–96)	
	AA	889.5 (532–1566.5)		0.08 (0.06–0.12)		510.5 (261.3–592.5)		79.5 (47.5–86)	
	GA + AA	745 (594.5–1039.5)	P_dom_ = 0.854	0.07 (0.05–0.10)	P_dom_ = 0.170	534.5 (458–641.8)	P_dom_ = 0.144	85 (71–92.8)	P_dom_ = 0.221
*CARD8* rs2043211	AA	731 (565–869)	0.451	0.06 (0.05–0.07)	0.663	650 (469–875)	0.134	90 (76–123.5)	0.147
	AT	742.5 (563.3–977.8)		0.06 (0.05–0.08)		603.5 (478.3–914)		94.5 (70.5–122.8)	
	TT	785 (721–1277)		0.08 (0.04–0.10)		470 (320–571)		68 (50–87)	
	AT + TT	758 (570–1005)	P_dom_ = 0.437	0.06 (0.05–0.08)	P_dom_ = 0.588	567 (404–898)	P_dom_ = 0.491	83 (61–117)	P_dom_ = 0.291
*NLRP3* rs35829419	CC	748 (572.5–948.5)	0.413	0.06 (0.05–0.08)	0.168	576 (461–879.5)	0.264	87 (70–119.5)	0.130
	CA+AA	591 (516–790.5)		0.04 (0.04–0.05)		709 (663–1003.5)		109 (103–149.5)	
*GSTP1* rs1695	AA	759 (576–936)	0.883	0.06 (0.05–0.08)	0.416	562.5 (468.5–890.8)	0.846	87.5 (69.5–123.8)	0.550
	AG	758.5 (572.3–984.8)		0.06 (0.04–0.08)		615 (403.8–980)		95.5 (67.8–124.3)	
	GG	688.5 (557.3–1010.5)		0.06 (0.06–0.08)		616.5 (467.8–788)		80 (72.3–98)	
	AG + GG	742.5 (564.5–981.5)	P_dom_ = 0.822	0.06 (0.05–0.08)	P_dom_ = 0.583	615 (461–876.5)	P_dom_ = 0.855	90 (70.5–120.8)	P_dom_ = 0.779
*GSTP1* rs1138272	CC	745 (552–919.8)		0.06 (0.05–0.08)		601.5 (470–880.3)		88.5 (70.8–120.8)	
	CT + TT	790 (674.5–1086.3)	P_dom_ = 0.224	0.07 (0.06–0.10)	P_dom_ = 0.294	572.5 (359–887.8)	P_dom_ = 0.650	86.5 (60.8–118.3)	P_dom_ = 0.944
*NOS1* rs2293054	AA	753 (584.3–1042.3)	0.738	0.06 (0.05–0.09)	0.420	569 (403.3–891.5)	0.670	86.5 (63.3–121.5)	0.914
	AG	765 (549.8–907)		0.06 (0.05–0.07)		605 (506.8–880.3)		90.5 (74.3–120)	
	GG	642.5 (571.3–981.5)		0.07 (0.05–0.07)		665 (312.8–936.3)		87.5 (55–120.5)	
	AG + GG	736 (563.3–907)	P_dom_ = 0.670	0.06 (0.05–0.07)	P_dom_ = 0.299	605 (497.5–880.3)	P_dom_ = 0.450	90.5 (73.3–120)	P_dom_ = 0.950
*NOS1* rs2682826	GG	734.5 (546.8–977.8)	0.371	0.06 (0.05–0.08)	0.731	615 (358.8–880.3)	0.204	91.5 (58–121.5)	0.261
	GA	770 (658–1005)		0.06 (0.05–0.08)		567 (470–782)		83 (70–107)	
	AA	602 (552–742.5)		0.06 (0.05–0.07)		911 (499–1152)		122 (76.5–144)	
	GA + AA	753 (611–947.3)	P_dom_ = 0.728	0.06 (0.05–0.08)	P_dom_ = 0.745	573.5 (473.3–894.3)	P_dom_ = 0.606	85.5 (74–120)	P_dom_ = 0.712
*KEAP1* rs1048290	GG	795 (578.5–1031)	0.222	0.06 (0.06–0.09)	0.547	549 (436.5–875)	0.723	93 (64.5–123.5)	0.957
	GC	711 (534–938.5)		0.06 (0.05–0.07)		617 (464.5–902.5)		88 (73–118)	
	CC	767 (656.8–975)		0.06 (0.04–0.08)		543 (455.5–885.3)		84 (74.5–119.8)	
	GC + CC	725 (570–941)	P_dom_ = 0.250	0.06 (0.05–0.08)	P_dom_ = 0.331	601 (461–894)	P_dom_ = 0.908	87 (74–117)	P_dom_ = 0.986
*KEAP1* rs9676881	GG	797.5 (580.3–1010.5)	0.188	0.06 (0.06–0.08)	0.565	581 (453.3–873.5)	0.735	91 (66.3–123.3)	0.959
	GA	703 (532–947.3)		0.06 (0.05–0.07)		609 (462.8–906.8)		87.5 (72.5–120)	
	AA	767 (656.8–975)		0.05 (0.04–0.08)		543 (455.5–885.3)		84 (74.5–119.8)	
	GA + AA	723 (562.3–944.8)	P_dom_ = 0.218	0.06 (0.04–0.08)	P_dom_ = 0.349	597.5 (461–895)	P_dom_ = 0.947	87 (73.5–118.3)	P_dom_ = 0.996
*NFE2L2* rs6706649	CC	765 (576.8–1054.5)	0.235	0.06 (0.05–0.08)	0.940	573.5 (416.5–878.8)	0.329	85.5 (62.8–116.3)	0.223
	CT	690 (531.3–817.5)		0.06 (0.04–0.08)		736 (486–942)		103.5 (74.8–131)	
	TT	790 (758–822)		0.06 (0.06–0.06)		534.5 (525–544)		83.5 (81–86)	
	CT + TT	714.5 (539.3–815.3)	P_dom_ = 0.143	0.06 (0.05–0.07)	P_dom_ = 0.733	669.5 (493–902.8)	P_dom_ = 0.210	97.5 (76–122.8)	P_dom_ = 0.117
*NFE2L2* rs6721961	GG	758 (578.5–915)		0.06 (0.05–0.08)		650 (493.5–916)		94 (74.5–122.5)	
	GT + TT	738 (539–1091)	P_dom_ = 0.827	0.06 (0.06–0.09)	P_dom_ = 0.313	473 (324–802)	P_dom_ = **0.020**	79 (50–114)	P_dom_ = 0.063
*NFE2L2* rs35652124	TT	817 (663–1125.5)	**0.031**	0.07 (0.06–0.09)	0.140	544 (430–868)	0.428	81 (69–117)	0.439
	TC	669 (538–795)		0.06 (0.04–0.07)		613 (496–894)		94 (76–123)	
	CC	794 (599.3–1055.3)		0.06 (0.05–0.11)		610.5 (430.8–913.8)		85 (63.3–111.5)	
	TC + CC	711 (539–916)	P_dom_ = 0.053	0.06 (0.05–0.07)	P_dom_ = 0.097	613 (496–894)	P_dom_ = 0.219	93 (74–122)	P_dom_ = 0.304

Aβ: amyloid β; SNP: single-nucleotide polymorphism. Statistically significant results are printed in bold.

**Table 4:** Association of investigated polymorphisms with cognitive test scores among all patients with dementia and AD patients.

		Dementia		AD	
SNP	Genotype	MMSE	*p*	MMSE	*p*
*SOD2* rs4880	CC	21.5 (20–25.3)	0.703	21 (17.8–23.5)	0.062
	CT	25 (16–27)		16 (13–21.5)	
	TT	24 (19–26.5)		21 (17.5–26)	
	CT + TT	24.5 (16.3–26.8)	P_dom_ = 0.474	21 (17.8–23.5)	P_dom_ = 0.417
*CAT* rs1001179	CC	24 (19.8–26.3)	**0.022**	20 (16–24)	0.114
	CT	22 (13.5–25.5)		15 (13–23)	
	TT	28 (27–28.5)		/ ^a^	
	CT + TT	23 (14.3–26)	P_dom_ = 0.449	15 (13–23)	P_dom_ = 0.114
*GPX1* rs1050450	CC	24.5 (20–26)	0.849	21 (16–26)	0.411
	CT	23 (17–27)		20 (15–24)	
	TT	23 (15–26)		15.5 (13.5–21.3)	
	CT + TT	23 (16–26.3)	P_dom_ = 0.687	21 (16–26)	P_dom_ = 0.275
*IL1B* rs1143623	GG	24.5 (20–26)	0.057	21 (16.5–25.5)	0.231
	GC	25 (17–27)		16 (13.5–22.8)	
	CC	21 (13–23)		16 (12–22.5)	
	GC + CC	22.5 (15–26)	P_dom_ = 0.337	21 (16.5–25.5)	P_dom_ = 0.087
*IL1B* rs16944	TT	21.5 (14.5–25.3)	0.213	18.5 (12.5–23.8)	0.879
	TC	26 (19.3–27)		19.5 (15–25.5)	
	CC	24 (18.5–26)		20 (16–23.5)	
	TC + CC	24 (19.3–26.8)	P_dom_ = 0.127	20 (15.3–24)	P_dom_ = 0.631
*IL1B* rs1071676	GG	25 (20–26)	0.440	20.5 (16.3–24)	**0.004**
	GC	21.5 (14.3–26.8)		14.5 (12.3–17)	
	CC	21.5 (18.8–25.5)		21 (17.5–26)	
	GC + CC	21.5 (15–26.3)	P_dom_ = 0.200	20 (15–24)	P_dom_ = 0.820
*MIR146A* rs2910164	GG	25 (19.3–26.8)	0.131	20 (16–24.8)	0.328
	GC	23.5 (17.8–26)		20.5 (14.8–24.5)	
	CC	18.5 (12–23.3)		14.5 (10–21.3)	
	GC + CC	22.5 (15.8–26)	P_dom_ = 0.317	18.5 (13.8–23.3)	P_dom_ = 0.338
*IL6* rs1800795	GG	21.5 (15–26)	0.151	19.5 (14.5–22.8)	0.391
	GC	25 (20.5–27)		21 (17–26)	
	CC	23 (16–25)		16 (13–23)	
	GC + CC	24 (20–26)	P_dom_ = 0.291	20.5 (16–24)	P_dom_ = 0.483
*TNF* rs1800629	GG	23 (17.5–26.5)	0.459	20 (15–23)	0.652
	GA	22 (16–25.5)		18 (14.75–24.5)	
	AA	26 (24.5–26)		/ ^b^	
	GA + AA	24 (18–26)	P_dom_ = 0.964	20 (16–24)	P_dom_ = 0.869
*CARD8* rs2043211	AA	22.5 (15.8–26)	0.249	20 (13–22)	0.399
	AT	25 (17.8–26)		20 (16–26)	
	TT	25.5 (20.8–28.3)		23 (16.3–26)	
	AT + TT	25 (19.3–26)	P_dom_ = 0.215	20 (16–26)	P_dom_ = 0.228
*NLRP3* rs35829419	CC	23.5 (17.3–26)	0.365	20 (15–23.5)	/
	CA	26 (26–26)		/ ^a^	
*GSTP1* rs1695	AA	22 (15–26)	0.410	16.5 (15–21.8)	0.308
	AG	25 (20–26)		21 (16–26)	
	GG	21 (19–26.5)		20 (14.3–24.8)	
	AG + GG	24.5 (20–26)	P_dom_ = 0.299	20.5 (16–26)	P_dom_ = 0.153
*GSTP1* rs1138272	CC	22 (16–26)		20 (15–22.3)	
	CT + TT	26.5 (24.3–27)	P_dom_ = **0.005**	24 (19.5–25.5)	P_dom_ = 0.144
*NOS1* rs2293054	AA	25 (20.3–27)	0.342	21 (15–24)	0.992
	AG	21 (17–26)		20 (15.3–23.3)	
	GG	25.5 (16.8–26.3)		18 (15.5–22)	
	AG + GG	21 (17–26)	P_dom_ = 0.192	20 (15–24)	P_dom_ = 0.970
*NOS1* rs2682826	GG	25 (16.5–26.5)	0.306	20 (14.8–25.3)	0.754
	GA	24 (19–26)		21 (15–24)	
	AA	20 (16.3–22.3)		20 (14.5–20.5)	
	GA + AA	22 (18.5–26)	P_dom_ = 0.591	20 (15.3–23.5)	P_dom_ = 0.980
*KEAP1* rs1048290	GG	23 (17.5–26.5)	**0.019**	19.5 (16–22.8)	**0.035**
	GC	22 (15–25)		16.5 (13–21.3)	
	CC	26 (24–28)		25.5 (20.3–26)	
	GC + CC	24 (18.5–26)	P_dom_ = 0.917	20 (13.8–24.3)	P_dom_ = 0.825
*KEAP1* rs9676881	GG	23 (17.5–26.5)	**0.019**	19.5 (16–22.8)	**0.035**
	GA	22 (15–25)		16.5 (13–21.3)	
	AA	26 (24–28)		25.5 (20.3–26)	
	GA + AA	24 (18.5–26)	P_dom_ = 0.917	20 (13.8–24.3)	P_dom_ = 0.825
*NFE2L2* rs6706649	CC	24.5 (18.3–26.8)		20 (15–24.5)	
	CT + TT	22 (16–26)	P_dom_ = 0.335	21 (15.5–23)	P_dom_ = 0.893
*NFE2L2* rs6721961	GG	23.5 (17.3–26)		20 (16–24)	
	GT + TT	25.5 (18.8–27)	P_dom_ = 0.391	15 (13–22)	P_dom_ = 0.272
*NFE2L2* rs35652124	TT	25 (21–27)	**0.030**	20 (15–24.5)	0.456
	TC	21 (15–26)		20 (14–23)	
	CC	23.5 (20.5–26.3)		21 (18.3–26)	
	TC + CC	21 (16–26)	P_dom_ = **0.024**	20 (15–23.5)	P_dom_ = 0.893

AD: Alzheimer’s disease; MMSE: The Mini-Mental State Exam. ^a^ No patients with this genotype were present in AD group; ^b^ since only 1 patient with this genotype was present in AD group, range could not be calculated. Statistically significant results are printed in bold.

## Data Availability

All the data are presented within the article and in Supplementary Material. Any additional information is available on request from the corresponding author.

## Supplementary Materials

The following supporting information can be downloaded at: https://www.mdpi.com/article/10.3390/antiox12020316/s1, Table S1: Genotype frequencies of selected polymorphisms; Table S2: Comparison of genotype frequencies among patients with different types of dementia; Table S3: Comparison of genotype frequencies among patients with AD and controls; Table S4: Association of investigated polymorphisms with cerebrospinal fluid biomarkers among patients with AD.
