# Supplementary material for: Genetic Polymorphisms in Oxidative Stress and Inflammatory Pathways as Potential Biomarkers in Alzheimer’s Disease and Dementia
Source: Antioxidants (Basel). 2023 Jan 29;12(2):316. doi: 10.3390/antiox12020316 (PMC9952323; doi:10.3390/antiox12020316)
Supplement: Supplementary file 1 [file antioxidants-12-00316-s001.zip › antioxidants-2090948-supplementary.pdf]

Table S1. Genotype frequencies of selected polymorphisms.

Gene	SNP	Role	Genotype	All subjects (N=150) N (%)	MAF (controls)	pHWE (controls)
SOD2	rs4880	p.Ala16Val	CC	35 (23.3)	0.52	0.624
			CT	75 (50)		
			TT	40 (26.7)		
CAT	rs1001179	c.-262C>T	CC	99 (66)	0.23	0.254
			CT	40 (26.7)		
			TT	11 (7.3)		
GPX1	rs1050450	p.Pro198Leu	CC	71 (47.3)	0.31	0.938
			CT	64 (42.7)		
			TT	15 (10)		
IL1B	rs1143623	c.-1560G>C	GG	85 (56.7)	0.25	0.446
			GC	46 (30.7)		
			CC	19 (12.7)		
	rs16944[a]	c.-598T>C	TT	23 (15.3)	0.69	0.938
			TC	58 (38.7)		
			CC	69 (46)		
	rs1071676	c.*505G>C	CC	87 (58)	0.26	0.057
			CG	49 (32.7)		
			GG	14 (9.3)		
MIR146A	rs2910164	n.60G>C	GG	89 (59.3)	0.22	0.964
			GC	51 (34)		
			CC	10 (6.7)		
IL6	rs1800795	c.-174G>C	GG	53 (35.3)	0.44	0.354
			GC	70 (46.7)		
			CC	27 (18)		
TNF	rs1800629	c.-308G>A	GG	114 (76)	0.17	0.482
			GA	31 (20.7)		
			AA	5 (3.3)		
CARD8	rs2043211	p.Cys10Ter	AA	69 (46)	0.27	0.673
			AT	66 (44)		
			TT	15 (10)		
NLRP3	rs35829419	p.Gln705Lys	CC	143 (95.3)	0.03	0.793

			CA	7 (4.7)		
			AA	0		
GSTP1	rs1695	p.Ile105Val	AA	64 (42.7)	0.32	0.793
			AG	66 (44)		
			GG	20 (13.3)		
	rs1138272	p.Ala114Val	CC	123 (82)	0.09	0.444
			CT	25 (16.7)		
			TT	2 (1.3)		
NOS1	rs2293054	p.Ile734=	AA	81 (54)	0.20	0.046
			AG	61 (40.7)		
			GG	8 (5.3)		
	rs2682826	c.*276G>A	GG	72 (48)	0.27	0.673
			GA	65 (43.3)		
			AA	13 (8.7)		
KEAP1	rs1048290	p.Leu471=	GG	50 (33.3)	0.37	0.054
			GC	77 (51.3)		
			CC	23 (15.3)		
	rs9676881	c.*548G>A	GG	50 (33.3)	0.38	0.035
			GA	77 (51.3)		
			AA	23 (15.3)		
NFE2L2	rs6706649	c. -767C>T	CC	118 (78.7)	0.12	0.012
			CT	27 (18)		
			TT	5 (3.3)		
	rs6721961	c.-733T>G	GG	113 (75.3)	0.11	0.316
			GT	36 (24)		
			TT	1 (0.7)		
	rs35652124	c.-769T>C	TT	60 (40)	0.42	0.274
			TC	65 (43.3)		
			CC	25 (16.7)		

[a]polymorphic allele more common in European populations

HWE: Hardy-Weinberg equilibrium; MAF: minor allele frequency; SNP: single-nucleotide polymorphism

Table S2. Comparison of genotype frequencies among patients with different types of dementia.

Gene	SNP	Genotype	AD N (%)	MCI N (%)	MCI (NOT AD) N (%)	P
SOD2	rs4880	CC	12 (22.2)	4 (28.6)	4 (20)	0.321
		CT	25 (46.3)	7 (50)	14 (70)	
		TT	17 (31.5)	3 (21.4)	2 (10)	
		CT+TT	42 (77.8)	10 (71.4)	16 (80)	P_{dom}= 0.873
CAT	rs1001179	CC	39 (72.2)	9 (64.3)	13 (65)	0.177
		CT	14 (25.9)	3 (21.4)	4 (20)	
		TT	1 (1.9)	2 (14.3)	3 (15)	
		CT+TT	15 (27.8)	5 (35.7)	7 (35)	P_{dom}= 0.719
GPX1	rs1050450	CC	27 (50)	5 (35.7)	10 (50)	0.295
		CT	21 (38.9)	6 (42.9)	10 (50)	
		TT	6 (11.1)	3 (21.4)	0	
		CT+TT	27 (50)	9 (54.3)	10 (50)	P_{dom}= 0.654
IL1B	rs1143623	GG	31 (57.4)	7 (50)	11 (55)	0.378
		GC	12 (22.2)	5 (35.7)	8 (40)	
		CC	11 (20.4)	2 (14.3)	1 (5)	
		GC+CC	23 (42.6)	7 (50)	9 (45)	P_{dom}= 0.910
	rs16944	TT	12 (22.2)	2 (14.3)	3 (15)	0.434
		TC	17 (31.5)	8 (57.1)	6 (30)	
		CC	25 (46.3)	4 (28.6)	11 (55)	
		TC+CC	42 (77.8)	12 (85.7)	17 (85)	P_{dom}= 0.798
	rs1071676	GG	32 (59.3)	8 (57.1)	10 (50)	0.790
		GC	17 (31.5)	6 (42.9)	8 (40)	
		CC	5 (9.3)	0	2 (10)	
		GC+CC	22 (40.8)	6 (42.9)	10 (50)	P_{dom}= 0.788
MIR146A	rs2910164	GG	31 (57.4)	6 (42.9)	14 (70)	0.242
		GC	18 (33.3)	8 (57.1)	4 (20)	
		CC	5 (9.3)	0	2 (10)	
		GC+CC	23 (42.6)	8 (57.1)	6 (30)	P_{dom}= 0.292
IL6	rs1800795	GG	23 (42.6)	6 (42.9)	3 (15)	0.152
		GC	22 (40.7)	7 (50)	14 (70)	
		CC	9 (16.7)	1 (7.1)	3 (15)	

		GC+CC	31 (57.4)	8 (57.1)	17 (85)	P_{dom}= 0.076
TNF	rs1800629	GG	44 (81.5)	12 (85.7)	16 (80)	0.443
		GA	9 (16.7)	1 (7.1)	2 (10)	
		AA	1 (1.9)	1 (7.1)	2 (10)	
		GA+AA	10 (18.6)	2 (14.2)	4 (20)	P_{dom}= 1.000
CARD8	rs2043211	AA	25 (46.3)	6 (42.9)	6 (30)	0.335
		AT	25 (46.3)	6 (42.9)	9 (45)	
		TT	4 (7.4)	2 (14.3)	5 (25)	
		AT+TT	29 (54.7)	8 (57.2)	14 (70)	P_{dom}= 0.463
NLRP3	rs35829419	CC	52 (96.3)	13 (92.9)	20 (100)	0.513
		CA	2 (3.7)	1 (7.1)	0	
GSTP1	rs1695	AA	22 (40.7)	6 (42.9)	8 (40)	1.000
		AG	23 (42.6)	6 (42.9)	9 (45)	
		GG	9 (16.7)	2 (14.3)	3 (15)	
		AG + GG	32 (59.3)	8 (57.2)	12 (60)	P_{dom}= 1.000
	rs1138272	CC	47 (87)	10 (71.4)	15 (75)	0.179
		CT	7 (13)	3 (21.4)	4 (20)	
		TT	0	1 (7.1)	1 (5)	
		CT + TT	7 (13)	4 (28.5)	5 (25)	P_{dom}= 0.254
NOS1	rs2293054	AA	23 (42.6)	8 (57.1)	13 (65)	0.359
		AG	26 (48.1)	4 (28.6)	6 (30)	
		GG	5 (9.3)	2 (14.3)	1 (5)	
		AG+GG	31 (57.4)	6 (42.9)	7 (35)	P_{dom}= 0.190
	rs2682826	GG	24 (44.4)	5 (35.7)	11 (55)	0.349
		GA	22 (40.7)	9 (64.3)	8 (40)	
		AA	8 (14.8)	0	1 (5)	
		GA+AA	30 (55.5)	9 (64.3)	9 (45)	P_{dom}= 0.540
KEAP1	rs1048290	GG	18 (33.3)	4 (28.5)	7 (35)	1.000
		GC	25 (46.3)	7 (50)	9 (45)	
		CC	11 (20.4)	3 (21.4)	4 (20)	
		GC + CC	36 (66.7)	10 (71.4)	13 (65)	P_{dom}= 1.000
	rs9676881	GG	19 (35.2)	4 (28.5)	7 (35)	0.996
		GA	24 (44.4)	7 (50)	9 (45)	
		AA	11 (20.4)	3 (21.4)	4 (20)	

		GA + AA	35 (64.8)	10 (71.4)	13 (65)	P_{dom}= 0.949
NFE2L2	rs6706649	CC	39 (72.2)	11 (78.6)	18 (90)	0.312
		CT	14 (25.9)	2 (14.3)	2 (10)	
		TT	1 (1.9)	1 (7.1)	0	
		CT + TT	15 (27.8)	3 (21.4)	2 (10)	P_{dom}= 0.260
	rs6721961	GG	43 (79.6)	10 (71.4)	12 (60)	0.291
		GT	10 (18.5)	4 (28.5)	8 (40)	
		TT	1 (1.9)	0	0	
		GT + TT	11 (20.4)	4 (28.5)	8 (40)	P_{dom}= 0.241
	rs35652124	TT	17 (31.5)	10 (71.4)	10 (50)	**0.043**
		TC	30 (55.6)	3 (21.4)	6 (30)	
		CC	7 (12.9)	1 (7.1)	4 (20)	
		TC + CC	37 (68.5)	4 (28.5)	10 (50)	P_{dom}= **0.021**

AD: Alzheimer's disease; MCI: mild cognitive impairment; SNP: single-nucleotide polymorphism

Table S3. Comparison of genotype frequencies among patients with AD and controls.

Gene	SNP	Genotype	Controls N (%)	AD N (%)	OR (95 % CI)	P	OR_{adj} (95 % CI)	P_{adj}
SOD2	rs4880	CC	15 (24.2)	12 (22.2)	Reference		Reference	
		CT	29 (46.8)	25 (46.3)	1.078 (0.426-2.727)	0.875	1.295 (0.394-4.262)	0.670
		TT	18 (29)	17 (31.5)	1.181 (0.431-3.234)	0.747	1.545 (0.439-5.432)	0.498
		CT+TT	47 (75.8)	42 (77.8)	1.117 (0.47-2.655)	0.802	1.399 (0.473-4.142)	0.544
CAT	rs1001179	CC	38 (61.3)	39 (72.2)	Reference		Reference	
		CT	19 (30.6)	14 (25.9)	0.718 (0.315-1.634)	0.430	0.563 (0.194-1.64)	0.292
		TT	5 (8.1)	1 (1.9)	0.195 (0.022-1.746)	0.144	0.105 (0.006-1.914)	0.128
		CT+TT	24 (38.7)	15 (27.8)	0.609 (0.278-1.335)	0.215	0.465 (0.167-1.291)	0.141
GPX1	rs1050450	CC	29 (46.8)	27 (50)	Reference		Reference	
		CT	27 (43.5)	21 (38.9)	0.835 (0.385-1.812)	0.649	0.71 (0.26-1.937)	0.504
		TT	6 (9.7)	6 (11.1)	1.074 (0.309-3.738)	0.911	1.73 (0.28-10.688)	0.555
		CT+TT	33 (53.2)	27 (50)	0.879 (0.423-1.824)	0.729	0.828 (0.323-2.124)	0.694
IL1B	rs1143623	GG	36 (58.1)	31 (57.4)	Reference		Reference	
		GC	21 (33.9)	12 (22.2)	0.664 (0.282-1.563)	0.348	0.407 (0.133-1.242)	0.114
		CC	5 (8.1)	11 (20.4)	2.555 (0.8-8.159)	0.113	2.760 (0.561-13.571)	0.212
		GC+CC	26 (42)	23 (42.6)	1.027 (0.491-2.15)	0.943	0.728 (0.279-1.898)	0.516
	rs16944	TT	6 (9.7)	12 (22.2)	Reference		Reference	
		TC	27 (43.5)	17 (31.5)	0.315 (0.099-0.997)	**0.049**	0.206 (0.046-0.924)	**0.039**
		CC	29 (46.8)	25 (46.3)	0.431 (0.141-1.316)	0.140	0.426 (0.100-1.818)	0.249
		TC+CC	56 (90.3)	42 (77.8)	0.375 (0.13-1.081)	0.069	0.307 (0.078-1.201)	0.090
	rs1071676	CC	37 (59.7)	32 (59.3)	Reference		Reference	
		CG	18 (29)	17 (31.5)	1.092 (0.484-2.466)	0.832	0.945 (0.337-2.644)	0.914
		GG	7 (11.3)	5 (9.3)	0.826 (0.239-2.858)	0.763	0.613 (0.117-3.21)	0.562
		CG+GG	25 (40.3)	22 (40.8)	1.017 (0.484-2.139)	0.964	0.855 (0.331-2.208)	0.747
MIR146A	rs2910164	GG	38 (61.3)	31 (57.4)	Reference		Reference	
		GC	21 (33.9)	18 (33.3)	1.051 (0.478-2.311)	0.902	1.061 (0.385-2.919)	0.909
		CC	3 (4.8)	5 (9.3)	2.043 (0.452-9.229)	0.353	8.102 (0.893-73.5)	0.063
		GC+CC	24 (38.7)	23 (42.6)	1.175 (0.559-2.47)	0.671	1.334 (0.51-3.493)	0.557
IL6	rs1800795	GG	21 (33.9)	23 (42.6)	Reference		Reference	
		GC	27 (43.5)	22 (40.7)	0.744 (0.329-1.683)	0.478	0.572 (0.192-1.699)	0.314
		CC	14 (22.6)	9 (16.7)	0.587 (0.211-1.636)	0.308	0.355 (0.088-1.422)	0.143

		GC+CC	41 (66.1)	31 (57.4)	0.69 (0.325-1.466)	0.335	0.493 (0.181-1.348)	0.168
TNF	rs1800629	GG	42 (67.7)	44 (81.5)	Reference		Reference	
		GA	19 (30.6)	9 (16.7)	0.452 (0.184-1.111)	0.083	0.238 (0.069-0.823)	**0.023**
		AA	1 (1.6)	1 (1.9)	0.955 (0.058-15.758)	0.974	0.771 (0.002-374.434)	0.934
		GA+AA	20 (32.2)	10 (18.6)	0.477 (0.2-1.138)	0.095	0.246 (0.072-0.84)	**0.025**
CARD8	rs2043211	AA	32 (51.6)	25 (46.3)	Reference		Reference	
		AT	26 (41.9)	25 (46.3)	1.231 (0.577-2.627)	0.592	0.617 (0.221-1.723)	0.357
		TT	4 (6.5)	4 (7.4)	1.28 (0.291-5.631)	0.744	0.763 (0.119-4.88)	0.775
		AT+TT	30 (48.4)	29 (54.7)	1.237 (0.596-2.57)	0.568	0.638 (0.238-1.708)	0.371
NLRP3	rs35829419	CC	58 (93.5)	52 (96.3)	Reference		Reference	
		CA	4 (6.5)	2 (3.7)	0.558 (0.098-3.172)	0.510	0.553 (0.049-6.29)	0.633
GSTP1	rs1695	AA	28 (45.2)	22 (40.7)	Reference		Reference	
		AG	28 (45.2)	23 (42.6)	1.045 (0.477-2.292)	0.912	1.182 (0.426-3.274)	0.748
		GG	6 (9.7)	9 (16.7)	1.909 (0.59-6.177)	0.280	2.285 (0.516-10.124)	0.276
		AG + GG	34 (54.9)	32 (59.3)	1.198 (0.573-2.506)	0.632	1.376 (0.526-3.596)	0.515
	rs1138272	CC	51 (82.3)	47 (87)	Reference		Reference	
		CT	11 (17.7)	7 (13)	0.691 (0.247-1.929)	0.480	0.400 (0.104-1.542)	0.183
NOS1	rs2293054	AA	37 (59.7)	23 (42.6)	Reference		Reference	
		AG	25 (40.3)	26 (48.1)	1.673 (0.785-3.565)	0.182	1.949 (0.731-5.196)	0.182
		GG	0	5 (9.3)	/	**0.012***	/	/
		AG+GG	25 (40.3)	31 (57.4)	1.995 (0.951-4.183)	0.068	2.346 (0.893-6.166)	0.084
	rs2682826	GG	32 (51.6)	24 (44.4)	Reference		Reference	
		GA	26 (41.9)	22 (40.7)	1.128 (0.519-2.452)	0.761	0.910 (0.335-2.469)	0.853
		AA	4 (6.5)	8 (14.8)	2.667 (0.718-9.9)	0.143	1.518 (0.304-7.579)	0.611
		GA+AA	30 (48.4)	30 (55.5)	1.333 (0.641-2.773)	0.441	1.010 (0.394-2.589)	0.983
KEAP1	rs1048290	GG	21 (33.9)	18 (33.3)	Reference		Reference	
		GC	36 (58.1)	25 (46.3)	0.81 (0.36-1.822)	0.611	0.392 (0.127-1.205)	0.102
		CC	5 (8)	11 (20.4)	2.567 (0.75-8.785)	0.133	1.900 (0.374-9.648)	0.439
		GC + CC	41 (66.1)	36 (66.7)	1.024 (0.473-2.218)	0.951	0.553 (0.194-1.575)	0.267
	rs9676881	GG	20 (32.2)	19 (35.2)	Reference		Reference	
		GA	37 (59.7)	24 (44.4)	0.683 (0.303-1.537)	0.357	0.377 (0.124-1.152)	0.087
		AA	5 (8)	11 (20.4)	2.316 (0.677-7.919)	0.181	1.85 (0.365-9.376)	0.458
		GA + AA	42 (67.7)	35 (64.8)	0.877 (0.405-1.898)	0.739	0.531 (0.188-1.505)	0.234
NFE2L2	rs6706649	CC	50 (80.6)	39 (72.2)	Reference		Reference	

	CT	9 (14.5)	14 (25.9)	1.994 (0.782-5.086)	0.148	2.701 (0.806-9.049)	0.107
	TT	3 (4.8)	1 (1.9)	0.427 (0.043-4.269)	0.469	0.181 (0.011-2.948)	0.230
	CT + TT	12 (19.3)	15 (27.8)	1.603 (0.673-3.813)	0.286	1.808 (0.598-5.465)	0.294
rs6721961	GG	48 (77.4)	43 (79.6)	Reference		Reference	
	GT + TT	14 (22.6)	11 (20.4)	0.877 (0.36-2.137)	0.773	0.665 (0.219-2.016)	0.471
rs35652124	TT	23 (37.1)	17 (31.5)	Reference		Reference	
	TC	26 (41.9)	30 (55.6)	1.561 (0.689-3.537)	0.286	1.213 (0.421-3.492)	0.720
	CC	13 (21)	7 (12.9)	0.729 (0.24-2.216)	0.577	0.773 (0.195-3.063)	0.714
	TC + CC	39 (62.9)	37 (68.5)	1.284 (0.593-2.777)	0.526	1.063 (0.398-2.84)	0.904

*calculated using Fisher's exact test

Adj: adjusted for *APOE* status and age; AD: Alzheimer's disease; CI: confidence interval; OR: odds ratio; SNP: single-nucleotide polymorphism

Table S4. Association of investigated polymorphisms with cerebrospinal fluid biomarkers among patients with AD.

SNP	Genotype	Aβ42 (pg/ml)	P	Aβ42/40 ratio	P	Total tau (pg/ml)	P	pTau (pg/ml)	P
SOD2 rs4880	CC	727.5 (590.8-791.8)	0.575	0.06 (0.05-0.07)	0.234	733.5 (535.5-937.8)	0.777	95.5 (84.8-113)	0.880
	CT	711 (531-791.5)		0.06 (0.04-0.07)		782 (613-990)		98 (84.5-126.5)	
	TT	582 (534-742.5)		0.05 (0.03-0.06)		806 (513-1093)		100 (80-149)	
	CT+TT	663.5 (536-776.8)	P_{dom}=0.454	0.06 (0.04-0.06)	P_{dom}=0.482	792 (574.8-1014.8)	P_{dom}=0.662	99 (81-128)	P_{dom}=0.692
CAT rs1001179	CC	658 (530-770)		0.06 (0.04-0.07)		778 (549-1013)		98 (81-128)	
	CT+TT	725 (570-800)	P_{dom}=0.227	0.06 (0.05-0.06)	P_{dom}= 0.575	763 (576-969)	P_{dom}= 0.946	97 (81-117)	P_{dom}=0.395
GPX1 rs1050450	CC	695 (539-785)	0.285	0.05 (0.04-0.07)	0.955	748 (571-967)	0.461	98 (81-128)	0.678
	CT	669 (520.5-753.5)		0.06 (0.05-0.06)		855 (625.5-1085.5)		110 (83-133.5)	
	TT	758.5 (627-820)		0.06 (0.05-0.06)		696.5 (436.8-945)		91 (67.8-128)	
	CT+TT	688 (538-770)	P_{dom}=0.762	0.06 (0.05-0.06)	P_{dom}=0.763	844 (549-1020)	P_{dom}=0.586	98 (79-124)	P_{dom}=0.762
IL1B rs1143623	GG	718 (570-799)	0.280	0.06 (0.05-0.07)	0.542	763 (571-1035)	0.811	97 (81-140)	0.901
	GC	645.5 (532.3-722)		0.06 (0.04-0.06)		811.5 (562-1032.5)		106 (84.8-128)	
	CC	602 (504-785)		0.05 (0.04-0.07)		750 (521-967)		98 (79-123)	
	GC+CC	638 (523-758)	P_{dom}=0.113	0.05 (0.04-0.06)	P_{dom}=0.269	802 (549-969)	P_{dom}=0.896	98 (84-125)	P_{dom}=0.979
IL1B rs16944	TT	574 (505.8-778.5)	**0.038**	0.05 (0.04-0.06)	0.102	683.5 (521.8-944.8)	0.305	97.5 (75.3-121.5)	0.196
	TC	638 (526.5-726)		0.05 (0.04-0.06)		821 (605-1224.5)		114 (89.5-145.5)	
	CC	748 (584.5-808.5)		0.06 (0.05-0.07)		748 (551-902.5)		95 (81-125)	
	TC+CC	701 (556.5-777.5)	P_{dom}=0.328	0.06 (0.05-0.07)	P_{dom}=0.580	780 (588.3-1036)	P_{dom}=0.349	98 (83.3-131)	P_{dom}=0.417
IL1B rs1071676	GG	655.5 (527-743)	0.061	0.05 (0.04-0.07)	0.205	756.5 (572.3-1029.5)	0.950	99 (82.5-134.8)	0.474
	GC	748 (534-794)		0.06 (0.05-0.06)		782 (537-994.5)		89 (81-115.5)	
	CC	799 (714.5-952)		0.06 (0.06-0.07)		719 (620-883)		97 (92.5-116.5)	
	GC+CC	753 (562-804.3)	P_{dom}=0.088	0.06 (0.06-0.06)	P_{dom}=0.199	780 (544.5-925.5)	P_{dom}=0.765	96 (81-114.8)	P_{dom}=0.260
MIR146A rs2910164	GG	669 (530-759)	0.540	0.06 (0.04-0.06)	**0.033**	763 (515-1151)	0.947	98 (79-140)	0.933
	GC	703 (579-804.3)		0.07 (0.06-0.07)		765 (599.3-925)		97 (85.5-123.5)	
	CC	653 (574.5-855.5)		0.05 (0.04-0.07)		844 (665.5-875)		98 (92.5-123.5)	
	GC+CC	695 (582-800)	P_{dom}=0.267	0.06 (0.05-0.07)	P_{dom}=**0.043**	782 (609-894)	P_{dom}=0.773	98 (88-123)	P_{dom}=0.875
IL6 rs1800795	GG	731 (523-785)	0.832	0.06 (0.05-0.06)	0.894	719 (521-967)	0.423	97 (79-123)	0.558
	GC	691.5 (539.8-758.3)		0.06 (0.04-0.07)		813.5 (594.8-1067)		103.5 (85.5-132.8)	
	CC	658 (538.5-855.5)		0.06 (0.03-0.08)		750 (555.5-953.5)		97 (86.5-143)	
	GC+CC	669 (539-759)	P_{dom}=0.618	0.06 (0.04-0.07)	P_{dom}=0.732	802 (601-1035)	P_{dom}=0.298	100 (87-128)	P_{dom}=0.282
TNF rs1800629	GG	701 (532.3-773)		0.06 (0.04-0.06)		813.5 (572.3-1018.3)		108.5 (81.8-128)	
	GA	669 (556.5-797.5)		0.06 (0.05-0.08)		617 (499-736)		92 (75.5-97.5)	

	GA+AA	678.5 (565.8-796.3)	P_{dom}=0.824	0.06 (0.05-0.08)	P_{dom}=0.187	613 (500.5-729)	P_{dom}=0.058	90.5 (76.3-97.3)	P_{dom}=0.086
CARD8 rs2043211	AA	688 (538.5-797)	0.742	0.06 (0.05-0.06)	0.560	782 (605-925)	0.980	98 (85.5-128)	0.919
	AT	602 (536.5-766)		0.06 (0.05-0.07)		750 (528-1016.5)		98 (79-131)	
	TT	740.5 (511.8-778.8)		0.04 (0.03-0.07)		667 (533.5-1616.5)		94 (71.3-208.3)	
	AT+TT	707 (536.5-767)	P_{dom}=0.515	0.06 (0.04-0.07)	P_{dom}=0.850	750 (528-1016.5)	P_{dom}=0.924	98 (79-131)	P_{dom}=0.735
NLRP3 rs35829419	CC	691.5 (540-773)	1.000	0.06 (0.05-0.06)	0.633	770.5 (554.5-968.5)	0.453	98 (81-127.3)	0.314
	CA	715.5 (441-990)		0.05 (0.04-0.06)		1003.5 (709-1298)		143.5 (97-190)	
GSTP1 rs1695	AA	655.5 (526-771)	0.648	0.06 (0.05-0.07)	0.066	734.5 (544.5-984)	0.429	94.5 (82.8-138.8)	0.125
	AG	721 (539-795)		0.05 (0.03-0.06)		821 (601-1039)		114 (87-128)	
	GG	638 (553.5-765)		0.06 (0.06-0.07)		748 (519.5-825)		96 (76.5-98)	
	AG + GG	701 (543.8-793.3)	P_{dom}=0.418	0.06 (0.04-0.06)	P_{dom}=0.328	792 (578.5-1002)	P_{dom}=0.725	99 (81-124.8)	P_{dom}=0.641
GSTP1 rs1138272	CC	658 (530-774)		0.06 (0.04-0.06)		778 (571-969)		98 (81-128)	
	CT + TT	695 (669-885)	P_{dom}=0.242	0.07 (0.05-0.07)	P_{dom}=0.241	617 (525-1039)	P_{dom}=0.632	98 (81-128)	P_{dom}=0.960
NOS1 rs2293054	AA	707 (504-774)	0.547	0.06 (0.04-0.06)	0.801	782 (594-1035)	0.542	110 (81-144)	0.514
	AG	716 (536-791)		0.06 (0.05-0.06)		679.5 (544.5-898.3)		95.5 (80-123.5)	
	GG	602 (553.5-666.5)		0.06 (0.05-0.07)		844 (665-1003)		98 (85.5-132.5)	
	AG+GG	688 (543-785)	P_{dom}=0.773	0.06 (0.05-0.06)	P_{dom}=0.827	748 (549-911)	P_{dom}=0.490	97 (81-125)	P_{dom}=0.319
NOS1 rs2682826	GG	588 (505.8-798)	0.237	0.06 (0.04-0.06)	0.818	776 (598.8-950.3)	0.344	99 (88.5-127)	0.415
	GA	736 (649.3-785.8)		0.06 (0.05-0.07)		748 (560-928.5)		94 (81-119.5)	
	AA	588.5 (547.5-707)		0.06 (0.04-0.07)		939 (578-1208.5)		125 (82.3-147.5)	
	GA+AA	703 (571.5-771)	P_{dom}=0.296	0.06 (0.05-0.07)	P_{dom}=0.642	770.5 (543-1018.5)	P_{dom}=0.767	97 (81-130.3)	P_{dom}=0.702
KEAP1 rs1048290	GG	671 (545.3-800.5)	0.286	0.06 (0.05-0.06)	0.348	750 (529.3-1050.8)	0.668	99 (82.3-128.8)	0.860
	GC	669 (521.5-728)		0.06 (0.04-0.07)		802 (597.5-968)		98 (84-128)	
	CC	747 (653-799)		0.05 (0.04-0.06)		617 (515-1020)		92 (77-128)	
	GC + CC	691.5 (532-758.8)	P_{dom}=0.430	0.06 (0.04-0.06)	P_{dom}=0.440	772.5 (580.5-968.5)	P_{dom}=0.898	97.5 (81-128)	P_{dom}=0.826
KEAP1 rs9676881	GG	748 (546-800)	0.168	0.06 (0.05-0.06)	0.339	722 (531-1013)	0.622	98 (84-125)	0.848
	GA	653.5 (517.3-724)		0.06 (0.04-0.07)		811.5 (595.8-968.5)		102.5 (82.5-128)	
	AA	747 (653-799)		0.05 (0.04-0.06)		617 (515-1020)		92 (77-128)	
	GA + AA	688 (530-758)	P_{dom}=0.265	0.06 (0.04-0.07)	P_{dom}=0.390	782 (576-969)	P_{dom}=0.807	98 (81-128)	P_{dom}=0.964
NFE2L2 rs6706649	CC	695 (546-788)		0.06 (0.04-0.06)		763 (571-969)		97 (81-128)	
	CT + TT	669 (511-748)	P_{dom}=0.311	0.06 (0.05-0.07)	P_{dom}=0.373	778 (525-1035)	P_{dom}=0.794	110 (81-155)	P_{dom}=0.445
NFE2L2 rs6721961	GG	695 (546-788)		0.06 (0.05-0.07)		782 (601-1020)		98 (87-128)	
	GT + TT	653 (513-747)	P_{dom}=0.361	0.06 (0.03-0.06)	P_{dom}=0.756	594 (474-821)	P_{dom}=0.063	88 (79-128)	P_{dom}=0.259
	TT	718 (572.5-808)	0.693	0.06 (0.05-0.07)	0.349	782 (563-1003)	0.928	98 (81-132.5)	0.940

NFE2L2 rs35652124	TC	655.5 (536-771)		0.06 (0.04-0.06)		770.5 (544.5-938.3)		99 (83.3-125)	
	CC	759 (523-788)		0.06 (0.05-0.06)		748 (571-1013)		96 (81-125)	
	TC + CC	658 (534-772)	P_{dom}=0.451	0.06 (0.04-0.06)	P_{dom}=0.162	763 (560-991)	P_{dom}=0.801	98 (82.5-124.5)	P_{dom}=0.963

Aβ: amyloid β; AD: Alzheimer's disease; SNP: single-nucleotide polymorphism
